# Fe_3_O_4_@C Matrix with Tailorable Adsorption Capacities for Paracetamol and Acetylsalicylic Acid: Synthesis, Characterization, and Kinetic Modeling

**DOI:** 10.3390/molecules24091727

**Published:** 2019-05-03

**Authors:** Elena-Alina Moacă, Ciprian-Valentin Mihali, Ioana-Gabriela Macaşoi, Roxana Racoviceanu (Băbuţă), Codruţa Şoica, Cristina-Adriana Dehelean, Cornelia Păcurariu, Sorin Florescu

**Affiliations:** 1 “Victor Babeș” University of Medicine and Pharmacy Timișoara, Faculty of Pharmacy, Eftimie Murgu Square no.2, Timișoara RO-300041, Romania; alina.moaca@umft.ro (E.-A.M.); macasoi.ioana@umft.ro (I.-G.M.); codrutasoica@umft.ro (C.Ş.); cadehelean@umft.ro (C.-A.D.); 2 Politehnica University Timişoara, Faculty of Industrial Chemistry and Environmental Engineering, Victoriei Square no.2, Timişoara RO-300006, Romania; cornelia.pacurariu@upt.ro; 3 “Vasile Goldiş” Western University of Arad, The Institute of Life Sciences no.86, Liviu Rebreanu Street, RO-310414 Arad, Romania; mihaliciprian@yahoo.com; 4 “Victor Babeș” University of Medicine and Pharmacy Timișoara, Faculty of Medicine, Eftimie Murgu Square no.2, Timișoara RO-300041, Romania; florescusorin@yahoo.com

**Keywords:** Fe_3_O_4_@C matrix, combustion method, magnetic properties, adsorption, kinetics, isotherms

## Abstract

In this study Fe_3_O_4_@C matrix was obtained by combustion method and used hereafter as adsorbent for paracetamol and acetylsalicylic acid removal from aqueous solutions. The Fe_3_O_4_@C matrix was characterized by electronic microscopy, X-ray diffraction, thermal analysis, Fourier-transform infrared spectroscopy, and magnetic measurements. Two kinetic models of pseudo first-order and pseudo-second-order for both paracetamol and acetylsalicylic acid were studied. The experimental data were investigated by Langmuir, Freundlich, and Redlich–Peterson adsorption isotherm models. The adsorption followed the Redlich–Peterson and pseudo-second-order models with correlation coefficients R^2^ = 0.98593 and R^2^ = 0.99996, respectively, for the adsorption of paracetamol; for the acetylsalicylic acid, the adsorption followed the Freundlich and pseudo-second-order model, with correlation coefficients R^2^ = 0.99421 and R^2^ = 0.99977, respectively. The equilibrium was quickly reached after approximately 1h for the paracetamol adsorption and approximately 2h for acetylsalicylic acid adsorption. According to the Langmuir isotherm, the maximum adsorption capacity of the magnetic matrix was 142.01 mg·g^−1^ for the retention of paracetamol and 234.01 mg·g^−1^ for the retention of acetylsalicylic acid. The benefits of using the Fe_3_O_4_@C matrix are the low cost of synthesis and its easy and fast separation from solution by using an NdBFe magnet.

## 1. Introduction

Population development over the years had led to industrial progress [1,2] accompanied by undesired consequences, such as the negative polluting effects. These changes could not go for long untraced by the environment and humans. The pollution-related problems emerged years ago and continue to persist due to the contamination found in soil [3], air [4], and water [5]; therefore, solving these problems became an urgent matter.

Industry-related pollutants affect water in particular (ground water and surface water) frequently through untreated effluents, thus altering their properties and qualities [6,7]. Each class of pollutants requires a specific and proper removal procedure. The existent studies demonstrate that removal of dangerous pollutants has become a matter of great interest for many researchers [8,9]. There are many techniques that can be applied in order to extract the water pollutants, such as coagulation [10] and electrocoagulation [11], reverse osmosis [12], photocatalytic degradation [13], and adsorption [14,15,16]. These special procedures gained relevance because current water cleaning treatments were not efficient enough for either the elimination or the degradation of pollutants; thus the compounds could persist in the drinking water and could lead to health risks.

The pharmaceutical industry provides a multitude of drugs for human and veterinary use, consisting of chemical entities with biological activity that may affect living organisms; therefore, it is considered an important toxic pollutant generator for the ecosystem [17] and for humans as well. When the pharmaceutical compounds reach water (wastewater, rivers, and oceans), they affect the aquatic species [18] and life itself. Among the most important pollution sources, the untreated hospital and industrial effluents [19,20] and the improper disposal of used or expired pharmaceuticals [21] can be mentioned.

Paracetamol and acetylsalicylic acid exhibit antipyretic and analgesic properties and both are frequently used for the treatment of low to medium pain; in addition, acetylsalicylic acid acts as an anti-inflammatory agent. Despite their well-known benefits for human health, once they reach the wastewater they become toxic pollutants [14]. Negative effects are firstly experienced by the aquatic species and can be devastating. These chemical compounds or their degradation byproducts may persist and even accumulate after the usual cleaning treatments applied to the wastewater. The distribution of the drinking water containing such pollutants to the greater population may have dreadful results. This is why researchers struggled to find highly efficient procedures to remove these substances from waters [11,15,16,22,23,24]. 

Adsorption is a well-known and efficient technique used for the elimination of pollutants, in which the adsorbent may vary from carbon nanotubes [8] and polymeric materials [25] to various oxides [26], natural clay [27], or composite materials [28]. The adsorption process usually involves multistep filtration, long waiting time, and filtration materials [24,29]. Activated carbon is known as a very good adsorbent due to its unique porous texture and high adsorption capacity [14,27], while magnetic materials [29,30] have the advantage of fast magnetic separation; the only material used for separation in this case is an external magnet. Fast magnetic separation has significant economic impact, while providing high separation efficiency.

The activated charcoal is the most frequently used adsorbent, including the in vivo and in vitro studies. It was successfully used in drugs overdose as gastrointestinal adsorbent [31]. Çalişkan Salihi studied the adsorption of metamizol sodium on activated charcoal in simulated intestinal and gastric fluids. The author found that the maximum adsorption capacity was higher in the case of simulated gastric fluid as against intestinal fluid. Jain et al. [32] reported the adsorption of an antiviral drug onto activated charcoal; they found that the removal efficiency was 80–98% depending on the antiviral drug concentration, pH, and temperature.

In industry, activated charcoal is mainly used in wastewater treatment due to its versatility, large surface area, high adsorption capacity, and porosity. In order to achieve high removal efficiency, the activated carbon must develop a chemical interaction with the adsorbate [16,33].

Different methods can be approached for the synthesis of Fe_3_O_4_ such as coprecipitation, thermal decomposition, laser pyrolysis, and sol–gel and hydrothermal procedures [34,35,36,37,38]. A synthetic method that became widely used over the past few years is the combustion method, which involves the reaction between an oxidizing and reducing agent. The benefits of this method are short preparation time, the low cost of raw materials, and its ecofriendliness; in addition, an important aspect is the reduced energy consumption due to the one-step combustion process, which does not require any further thermal treatment [39].

The aim of this study was the synthesis of the Fe_3_O_4_@C matrix by the solution combustion method and the investigation of its adsorption capacity for the removal of paracetamol and acetylsalicylic acid from aqueous solution. The use of the Fe_3_O_4_@C matrix combines the advantages of magnetic separation with the benefits of a porous material and allows the achievement of a highly efficient adsorbent material. To the best of our knowledge, this is the first study focusing on the use of a magnetic matrix in the removal of both drugs from aqueous solution.

## 2. Results and Discussion

### 2.1. XRD Characterization of Fe_3_O_4_@C Matrix

**Figure 1:**
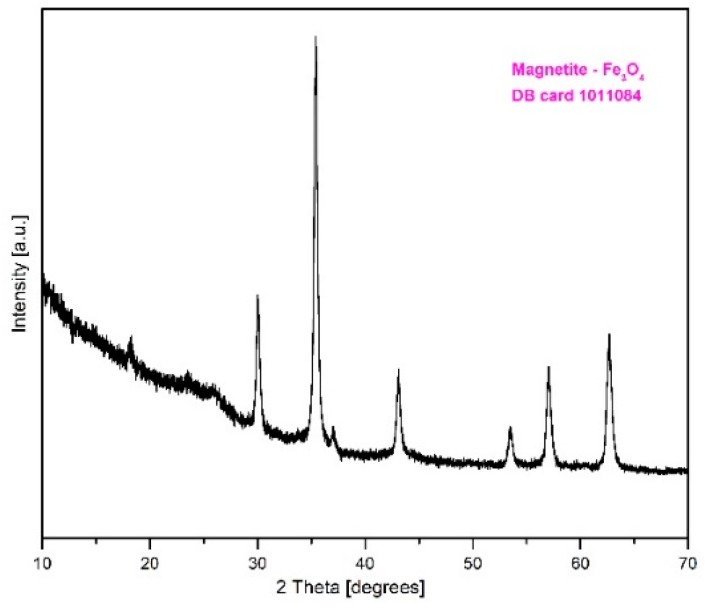
XRD pattern of the Fe_3_O_4_@C matrix prepared by combustion synthesis.

The XRD pattern (Figure 1) revealed that the analyzed sample contains only magnetite (Fe_3_O_4_) as single-phase according to the DB card 1011084; also, the active carbon is present in an amorphous phase which is not evidenced on the XRD pattern.

### 2.2. Magnetic Properties of Fe_3_O_4_@C Matrix

**Figure 2:**
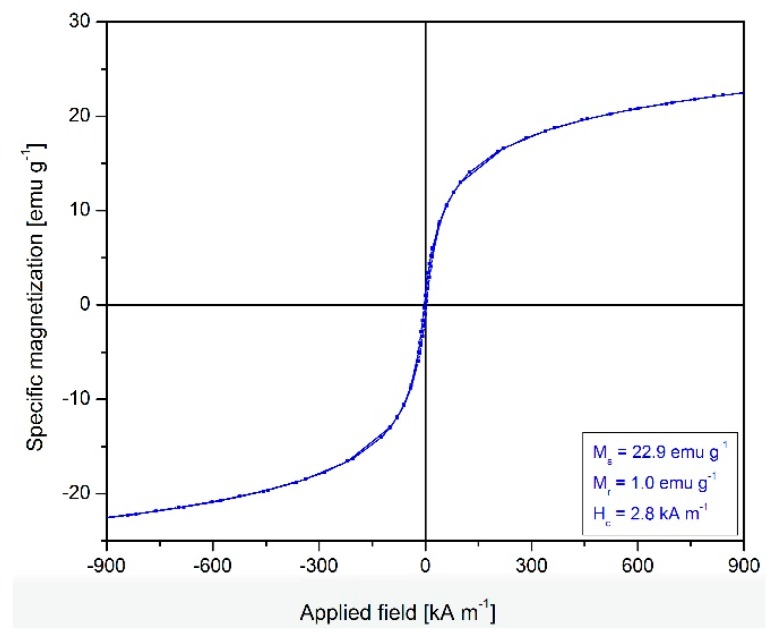
Magnetic hysteresis curve of the Fe_3_O_4_@C matrix.

Figure 2 shows the magnetic hysteresis loop of the Fe_3_O_4_@C matrix. The superparamagnetic behavior involves low values of remnant magnetization (Mr) and coercive field (Hc); the synthesized magnetic matrix is near to superparamagnetic behavior. Due to the fact that the Fe_3_O_4_@C matrix does not reach saturation over the applied magnetic field (H), saturation magnetization, Ms, was calculated by using the linear representation of magnetization versus 1/H and the extrapolation of 1/H→0.

The presence of carbon in the magnetic matrix led to the decrease of the saturation magnetization (Ms = 22.9 emu·g^−1^) when compared to the saturation magnetization of bulk magnetite (Ms = 84 emu·g^−1^) [40]. It is well known that a high magnetization value is correlated with larger particles; this observation is congenial with our results regarding the saturation magnetization and the matrix size evaluated by transmission electron microscopy Furthermore, our results are in agreement with previously reported literature data [41,42]. 

### 2.3. Thermal Analysis of Fe_3_O_4_@C Matrix

**Figure 3:**
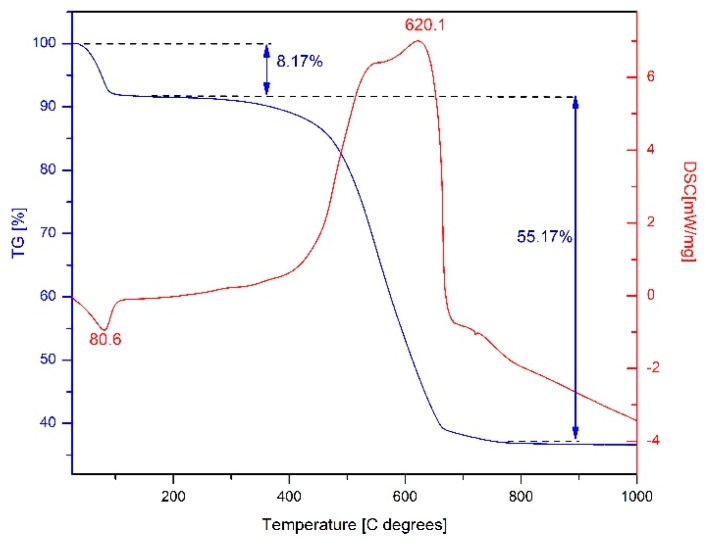
TG-DSC curves of the Fe_3_O_4_@C matrix.

The thermogravimetric (TG) and differential scanning calorimetry (DSC) curves of the Fe_3_O_4_@C matrix prepared by combustion method are depicted in Figure 3.

Thermal analysis revealed a total mass loss of 63.34% in two stages. In the first stage, the sample loses 8.17% of the total mass, and an endothermic process can be noticed with a maximum at 80.6 °C, attributed to the water removal. The second stage is wider, located between 300 °C and 650 °C, with a maximum at 620.1 °C; within this interval the sample loses 55.17% of the total mass and shows a strong exothermic effect assigned to the oxidation of the carbon content present in the sample.

The thermal analysis of our sample revealed neither the exothermic effect at ~212 °C characteristic to the magnetite oxidation to maghemite accompanied with mass growth on TG curve, nor the exothermic effect at ~500 °C without mass loss attributed to the maghemite transition to hematite. This behavior can be explained by the fact that the magnetite is embedded in a high content of carbon which protects it against oxidation to maghemite.

### 2.4. FTIR Investigations of Fe_3_O_4_@C Matrix

**Figure 4:**
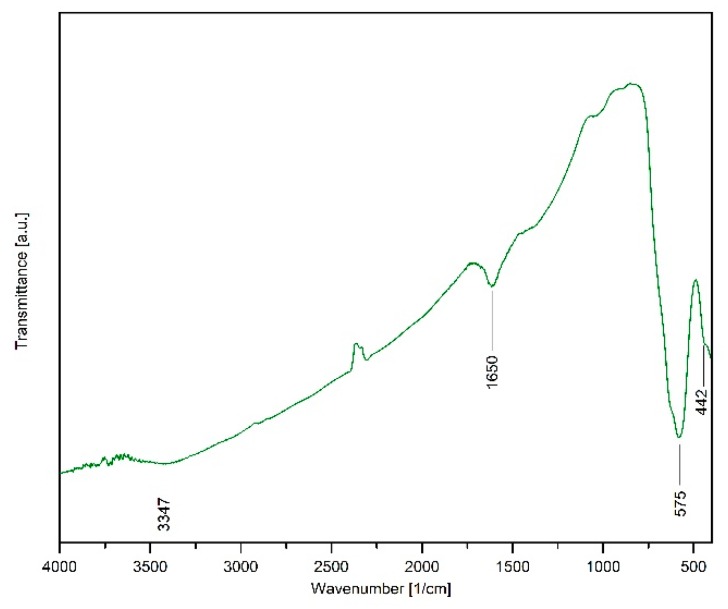
FTIR spectrum of the Fe_3_O_4_@C matrix.

The analysis of the FTIR spectrum (Figure 4) reveals the presence of bands located at 575 cm^−1^ and 442 cm^−1^ corresponding to the stretching vibration of Fe-O from the Fe_3_O_4_@C matrix [43,44,45]. This result is in agreement with the XRD analysis that proved magnetite to be a single phase. In addition, the FTIR spectrum shows the presence of amorphous phases that cannot be identified by X-ray diffraction. The band located at 1650 cm^−1^ can be attributed to the C=O stretching vibration, while the band located at 3347 cm^−1^ can be assigned to the O–H stretching vibration. These bands characterize the organic residue present in the sample after the raw materials combustion, in particular, the combustion fuel.

### 2.5. Electron Microscopy Characterization of Fe_3_O_4_@C Matrix

**Figure 5:**
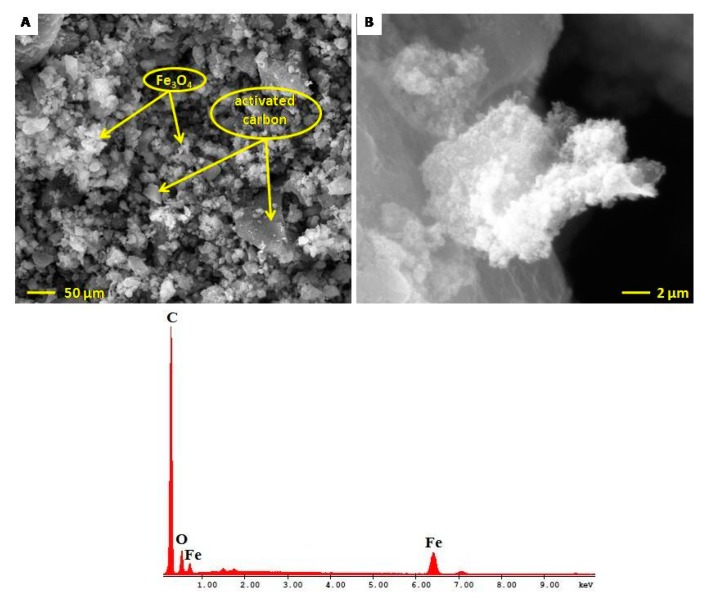
SEM-EDAX analysis of the Fe_3_O_4_@C matrix. (**A**) general overview—50 µm scale; (**B**) topology—2 µm scale; (**C**) EDAX.

**Figure 6:**
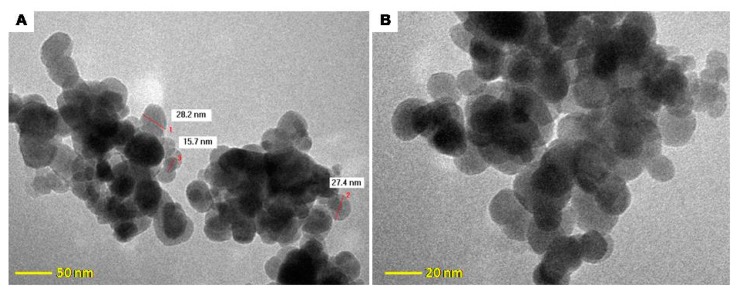
TEM micrograph of Fe_3_O_4_@C matrix. (**A**) magnification 220 Kx; (**B**) magnification 300 Kx.

The details regarding the morphology and ultrastructure of the samples were established by scanning electron microscopy - SEM (Figure 5) and transmission electron microscopy (Figure 6). The SEM general overview micrograph (Figure 5A) shows the Fe_3_O_4_@C matrix; one can notice that the magnetic matrix is characterized by differently sized spherical nanoparticles forming aggregates. Figure 5B shows the topology of the nanoparticles and their aggregates.

The energy dispersive X-ray analysis (EDAX) was employed to determine the elemental composition of the matrix (Table 1). The EDAX analysis showed that only C, O, and Fe were present in the Fe_3_O_4_@C matrix.

As shown in the TEM micrograph (Figure 6), the Fe_3_O_4_@C matrix consists of spherical nanoparticles with diameters of ~30 nm. The cluster size distribution was determined at higher magnification (220 Kx) in order to visualize the particles’ topology, and their organization as aggregates in the near-native solvent state was revealed (Figure 6A). At 300 Kx magnification (20-nm scale), the spherical form of the magnetic nanoparticles can be more accurately observed; they display multiple faceted surfaces with activated carbon deposits (Figure 6B).

### 2.6. Paracetamol and Acetylsalicylic Acid Adsorption on the Fe_3_O_4_@C Matrix

#### 2.6.1. Effect of the Initial Concentration on the Removal Efficiency

**Figure 7:**
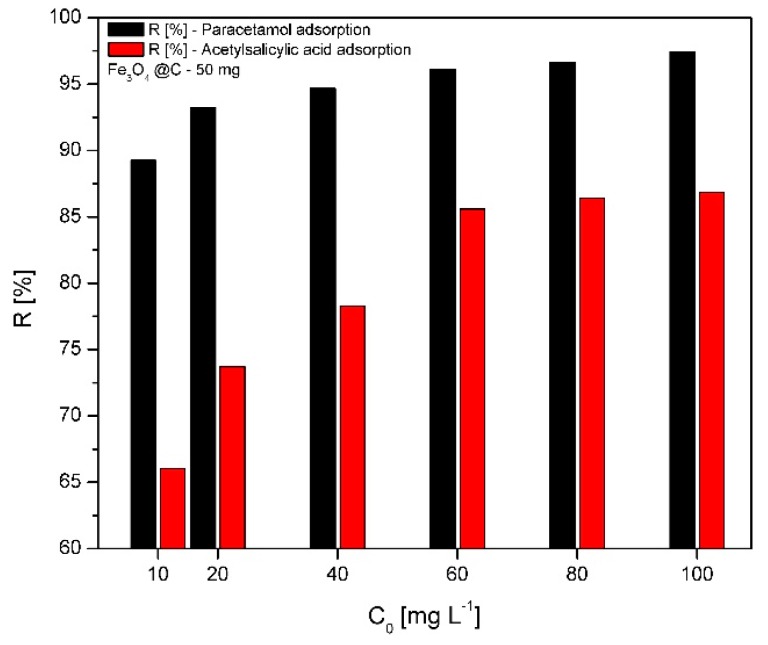
Removal efficiency of paracetamol and acetylsalicylic acid on the Fe_3_O_4_@C matrix.

The effect of the initial concentration of paracetamol and acetylsalicylic acid on the adsorption efficiency is shown in Figure 7. Using a Fe_3_O_4_@C matrix mass of 50 mg, the removal efficiency of paracetamol increases from 89% (at 10 mg/L) to 97% (at 100 mg/L). In the case of acetylsalicylic acid, the removal efficiency increases from 66% (at 10 mg/L) to 86% (at 100 mg/L). As one can notice, the adsorption efficiency is much higher for paracetamol than for acetylsalicylic acid; also, the adsorption efficiency is directly proportional to the drug concentration.

The removal efficiency of paracetamol from aqueous solution was higher than the removal of acetylsalicylic acid, probably due to the amino and phenol groups, which show affinity for the carbon shell present on the Fe_3_O_4_ surface. Our results are in perfect correlation with the data previously presented in the literature [46]. Ianoş et al. prepared magnetite nanoparticles embedded within a matrix of activated carbon using the combustion synthesis technique. The authors prepared magnetite/carbon nanocomposites with varied carbon ratio in order to remove phenol and p-chlorophenol from wastewater. They demonstrated that the removal efficiency of these organic pollutants (with very similar structure to the paracetamol—N-acetyl-para-aminophenol) from aqueous solution increased as the magnetite/carbon ratio decreased. They also tested the adsorption capacity of activated carbon in the removal of phenol and p-chlorophenol. Using a mass of 2 g/L activated carbon, the removal efficiency was 99%, while using a mass of 2 g/L nanocomposite (magnetite/carbon = 1:2), the removal efficiency was 94% [46]. 

Our synthesized compound (Fe_3_O_4_@C matrix) has an adsorption capacity of 97%, almost identical to the adsorption capacity of pure activated carbon, when 2 g/L of magnetic matrix (with mass ratio Fe_3_O_4_/C = 1:2 was used). Based on this result, we can state that the Fe_3_O_4_@C matrix can be successfully employed in the paracetamol and acetylsalicylic acid adsorption from wastewater.

The benefits of using the Fe_3_O_4_@C matrix instead of pure activated charcoal include the fast and easy magnetic separation instead of the centrifugation and filtration of activated charcoal and the possibility of its use in several desorption–adsorption cycles; pure active carbon can only be used once.

#### 2.6.2. Effect of Contact Time

**Figure 8:**
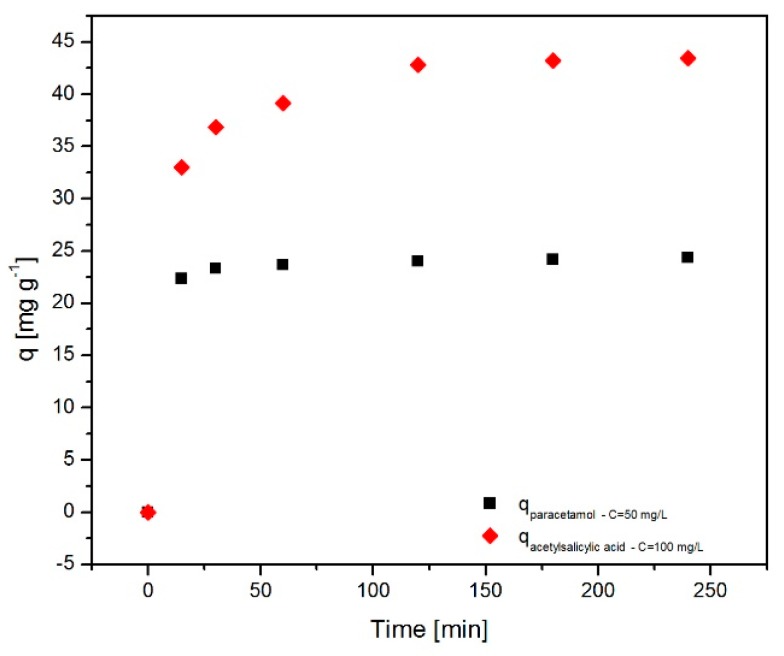
Adsorbed amount versus time for paracetamol and acetylsalicylic acid on the Fe_3_O_4_@C matrix.

Figure 8 shows the effect of the contact time on the adsorption of paracetamol and acetylsalicylic acid onto the Fe_3_O_4_@C matrix. One can notice that in the case of acetylsalicylic acid almost the entire drug amount is slowly adsorbed within 100 min, after which the system reaches equilibrium. For paracetamol, the adsorption process is very fast in the first 25 min, and then equilibrium is achieved. This behavior is due to the Fe_3_O_4_@C matrix surface, more precisely its carbon outer layer, which has numerous vacant sites that can be gradually occupied over time as a result of the adsorption process. It is also of note that the amount of drug adsorbed in the case of acetylsalicylic acid is higher than the adsorbed amount of paracetamol. This result is consistent and in perfect agreement with the adsorption efficiency (Figure 7), given the drug concentrations used in the experiment (C = 100 mg/mL acetylsalicylic acid; C = 50 mg/mL paracetamol).

#### 2.6.3. Kinetic Studies

**Figure 9:**
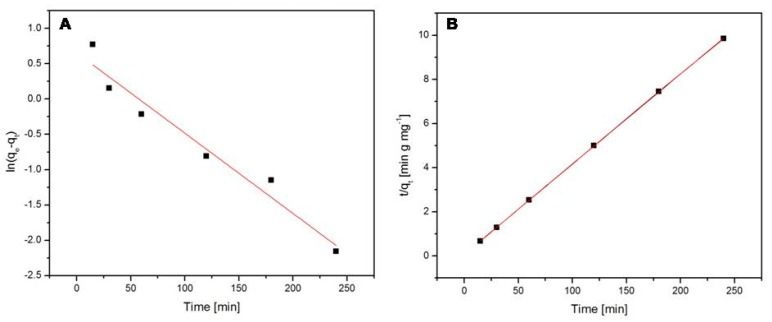
The pseudo-first-order (**A**) and the pseudo-second-order (**B**) models for paracetamol adsorption.

**Figure 10:**
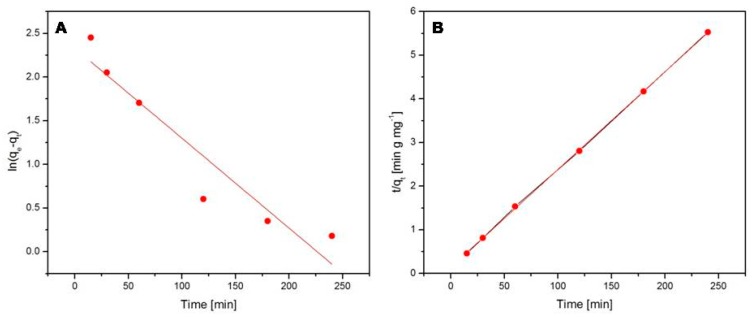
The pseudo-first-order (**A**) and the pseudo-second-order (**B**) models for acetylsalicylic acid adsorption.

The adsorption kinetics of paracetamol and acetylsalicylic acid onto the Fe_3_O_4_@C matrix were investigated using the pseudo-first-order (Equation (4)) and the pseudo-second-order kinetic models (Equation (6)). Figure 9 displays the pseudo-first-order model (Figure 9A) and the pseudo-second-order model (Figure 9B) of the paracetamol adsorption onto the Fe_3_O_4_@C matrix. Figure 10 shows the pseudo-first-order model (Figure 10A) and the pseudo-second-order model (Figure 10B) in the case of acetylsalicylic acid adsorption onto the Fe_3_O_4_@C matrix. The calculated values of k_1_, k_2_, q_e_, and R^2^ for paracetamol and acetylsalicylic acid adsorption are shown in Table 2.

The value of the correlation coefficient (Table 2) obtained for the pseudo-first-order model is lower than the one calculated for the pseudo-second-order model in both cases, which suggests that the paracetamol and acetylsalicylic acid kinetic adsorptions onto the Fe_3_O_4_@C matrix are more accurately described by the pseudo-second-order kinetic model.

#### 2.6.4. Adsorption Isotherms

**Figure 11:**
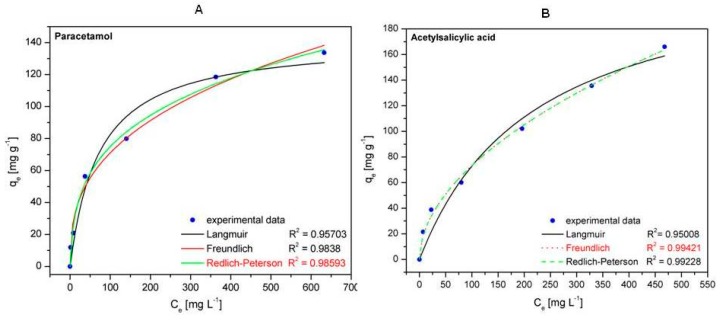
Isotherm plots for paracetamol (**A**) and acetylsalicylic acid (**B**) on the Fe_3_O_4_@C matrix.

The experimental data were investigated with Langmuir (Equation (7)), Freundlich (Equation (8)), and Redlich–Peterson (Equation (11)) adsorption isotherms (Figure 11). These isotherms provide significant information concerning the surface properties of the carbon matrix and the affinities between the Fe_3_O_4_@C matrix and the adsorbed drugs (paracetamol and acetylsalicylic acid, respectively), parameters of paramount importance for the adsorption process, and its efficiency.

The main parameters obtained from the fittings of the three equations and the corresponding correlation coefficients (R^2^) are listed in Table 3. By comparing the R^2^ values of the analyzed isotherms, one can notice that the Redlich–Peterson model is the most suitable to describe the paracetamol adsorption onto the Fe_3_O_4_@C matrix, while the Freundlich model is the most appropriate to describe the acetylsalicylic acid adsorption onto the Fe_3_O_4_@C matrix. From the literature [47] it is well known that the Redlich–Peterson is an empiric isotherm that combines the characteristics of both Langmuir and Freundlich models. This isotherm contains three parameters, one of which (β) is an exponent ranging between 0 and 1. When β = 1, the Redlich–Peterson model (Equation (11)) becomes the Langmuir isotherm (Equation (7)). Our results reveal that the adsorption of paracetamol onto the Fe_3_O_4_@C matrix follows a combination of the Langmuir and Freundlich isotherms, closer to the Langmuir model taking into account the value of β = 0.71299, which is near to β = 1.

Regarding the Freundlich isotherm, which describes the acetylsalicylic acid adsorption onto the Fe_3_O_4_@C matrix, the value of the exponent n also offers information about the adsorption process. A good adsorption process is characterized by values of exponent n between 2 and 10, according Hamdaoui and Naffrechoux, 2007 [48]. In our case, the value *n* = 1.91832 (Table 3) indicates that the matrix based on Fe_3_O_4_@C is an appropriate adsorbent for acetylsalicylic acid. 

Also, the maximum adsorption capacity of Fe_3_O_4_@C matrix resulting from the Langmuir isotherm is 234.01 mg g^−1^, which is higher than the other data previously reported in the literature [49,50,51].

Table 4 and Table 5 show data from the literature regarding the maximum adsorption capacity as well as the adsorption parameters concerning paracetamol and acetylsalicylic acid removal. One can notice that our synthesized magnetic matrix (Fe_3_O_4_@C) exhibits a very good adsorption capacity for the removal of paracetamol and acetylsalicylic acid. 

The Fe_3_O_4_@C matrix conjoins both the enhanced adsorption capacity and the high separation ability due to the magnetic properties of F_3_O_4_ nanoparticles. The magnetic separation is an unconventional technology, economic, and one-step solution because it does not involve extra materials and supplementary steps such as centrifugation and filtration. The magnetic separation took place instantaneously using an NdBFe magnet, thus reducing the manipulation time of the experiment. In addition, the as synthesized magnetic matrix comes with the advantage of a short adsorption time until reaching equilibrium.

## 3. Experimental Part

### 3.1. Preparation of Aqueous Solution Containing Drugs

Paracetamol (C_8_H_9_NO_2_—acetaminophen, N-acetyl-para-aminophenol (APAP)) is a member of the class of analgesic and antipyretic drugs widely used for the relief of severe/minor pain and as fever reducer. Acetylsalicylic acid (C_9_H_8_O_4_—2-acetoxybenzoic acid, aspirin) is considered an NSAID (nonsteroidal anti-inflammatory drug) due to its anti-inflammatory activity as well as analgesic and antipyretic properties. Paracetamol was acquired from Sigma-Aldrich and it was prepared an aqueous stock solution of 900 mg/L, pH = 3. Acetylsalicylic acid was achieved from Acros Organics and the aqueous stock solution prepared was of 800 mg/L, pH = 3. From each stock solution an additional six solutions were prepared, with concentrations between 10 and 900 mg/L for paracetamol and 10 and 800 mg/L for acetylsalicylic acid, in order to realize the adsorption isotherms. For the kinetic study and for the determination of the equilibrium time, it only one concentration for each drug solution was used: 50 mg/L for paracetamol and 100 mg/L for acetylsalicylic acid. 

In order to establish the removal efficiency, in case of both drugs, another six solutions were prepared with the following concentrations; 10, 20, 40, 60, 80, and 100 mg/L. 

### 3.2. Fe_3_O_4_@C Matrix Preparation

**Figure 12:**
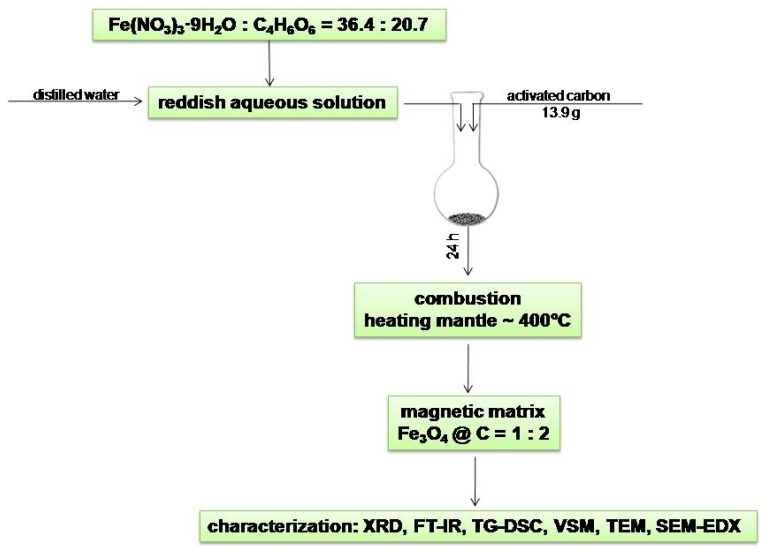
General preparation scheme of Fe_3_O_4_@C matrix.

A magnetic matrix consisting of iron oxide magnetic nanoparticles (magnetite—Fe_3_O_4_) and activated carbon [46] was prepared for the adsorption of the two analgesic drugs from aqueous solutions. Briefly, 36.4 g Fe(NO_3_)_3_·9H_2_O (Roth, Karlsruhe, Germany, 96%) and 20.7 g tartaric acid (C_4_H_6_O_6_) (Merck, Darmstadt, Germany, 99.5%) were dissolved in 50 mL distilled water and the resulting solution was then transferred into a round-bottom flask containing 13.9 g activated carbon. The mixture was kept for 24 h to impregnate and was afterwards placed inside a heating mantle at 400 °C so that the combustion reaction could take place (Figure 12).

The combustion reaction took place in the absence of air just to prevent the formation of γ-Fe_2_O_3_ instead of Fe_3_O_4_. The gases released after the combustion process were evacuated in a distilled water dish according to the method described by Ianoş and coworkers [39]. After gaseous emission stopped, the obtained magnetic black powder was washed several times with warm distilled water (60–80 °C) and then dried in an oven at 70 °C.

### 3.3. Fe_3_O_4_@C Matrix Characterization

The phase composition of the sample was investigated by X-ray diffraction (XRD) using a Rigaku Ultima IV instrument (Tokyo, Japan) operating at 40 kV and 40 mA. The XRD pattern was recorded using CuK_α_ radiation. The magnetic property of the sample was measured at room temperature by vibrating sample magnetometry using a VSM 880 ADE/DMS instrument (Massachusetts, USA). The heating behavior of the sample was studied within the range of 25 to 1000 °C by thermal analysis using a Netzsch STA 449 C instrument (Selb, Germany). The thermogravimetric (TG) and differential scanning calorimetry (DSC) curves were recorded using aluminum crucibles under artificial air flow of 20 mL/min at a heating rate of 10 °C/min. 

The FTIR spectrum of the sample was carried out using a Shimadzu Prestige-21 spectrometer (Duisburg, Germany) within the range of 400 to 4000 cm^−1^, using KBr pellets and a resolution of 4 cm^−1^. 

SEM-EDAX analysis was carried out with EDX detection on scanning electron microscope (SEM), using an EDAX detector (ZAF Quantification—Standardless, Element Normalized) with FEI Quanta 250 microscope (Eindhoven, Holland). SEM analysis parameters were HV mode, 30 kV, ETD (Everhart–Thornley detector for secondary electrons), with two magnification orders, one for a general overview of the image/measurements and another for higher surface topography for regions of interest. The identified chemical species were expressed in weight percent (Wt %) or atomic percent (At %).

### 3.4. Paracetamol and Acetylsalicylic Acid Adsorption Studies

The adsorption experiments for the removal of the drugs from aqueous solution were performed at 25 °C, using 2 g/L adsorbent mass, and various initial concentrations: 10–900 mg/L for paracetamol and 10–800 mg/L for acetylsalicylic acid solutions. In order to study the adsorption kinetics, 25 mL of both paracetamol and acetylsalicylic acid solutions (initial concentration—50 mg/L for paracetamol solution and 100 mg/L for acetylsalicylic acid solution) were mixed with 50 mg of Fe_3_O_4_@C matrix in an Erlenmeyer flask and continuously stirred at 250 rpm and controlled temperature of 25°C in an Environmental Shaker Incubator ES-20/60 BioSan (Riga, Latvia).

The recording started when the stirring began and six samples were collected between 15 min and 4 h. All adsorption assays were made in duplicate. After the magnetic separation, the concentrations of both paracetamol and acetylsalicylic acid solutions remaining in the Erlenmeyer flask were assessed with a UV–Vis spectrophotometer T70 UV/Vis Spectrometer, PG Instruments (Leicestershire, UK) at 202 nm for paracetamol and 226 nm for acetylsalicylic acid. The adsorbed amount (q_t_ [mg/g]) and the removal efficiency (R [%]) as a result of the adsorption process were determined according to Equations (1) and (2):

(1) $$q_{t}=\frac{C_{0}-C_{t}}{V}\times \;m_{ads}$$

(2) $$R=\;\frac{C_{0}-C_{e}}{C_{0}}\times 100$$

where *C*_0_—the initial concentrations of the paracetamol and acetylsalicylic acid solutions, respectively [mg/L]; *C_t_*—the solution concentrations at time t [mg/L]; *C_e_*—the solution concentrations at equilibrium [mg/L]; *V*—the volume of the aqueous solutions [L]; and *m_ads_*—the adsorbent mass [g].

The time needed to reach equilibrium was chosen based on the kinetic studies of Fe_3_O_4_@C matrix—3 h for both drugs. The kinetics of paracetamol and acetylsalicylic acid adsorption, respectively, onto the Fe_3_O_4_@C matrix was evaluated by means of two different models: the Lagergren pseudo-first-order [55] and pseudo-second-order, respectively [56]. 

The rate expression for the Lagergren pseudo-first-order is represented by the equation

(3) $$\frac{dq_{t}}{dt}=\;k_{1}\times \left(q_{e}-q_{t}\right)$$

where, *k*_1_—the adsorption rate constant of the pseudo-first-order [min^−1^]; *q*_e_—amount of solute adsorbed at equilibrium [mg/g]; and *q_t_*—amount of solute adsorbed at time t [mg/g].

The integration of Equation (3) between the limits *t* = 0 and *t* = *t*, with the values of *q_t_* = 0 and *q_t_* = *t*, respectively, gives the linear form (Equation (4)) of the Lagergren equation:

(4) $$\ln\left(q_{e}-q_{t}\right)=\;\ln q_{e}-\;k_{1}\times t$$

where, *q_e_*—amount of solute adsorbed at equilibrium [mg/g]; *q_t_*—amount of solute adsorbed at time *t* [mg/g].

By graphically representing the mathematical function a straight line will be obtained, from which one can calculate the adsorption rate constant of the pseudo-first-order (*k*_1_) by using the slope of the straight line. 

The rate expression for the pseudo-second-order is given by the equation [57]

(5) $$\frac{dq_{t}}{dt}=k_{2}\times {\left(q_{e}-q_{t}\right)}^{2}$$

where, *k*_2_—adsorption rate constant of the pseudo-second-order [g/mg·min].

By integrating the Equation (5) between the limits *t* = 0 and *t* = *t* and by using the values *q_t_* = 0 at *q_t_* = *t*, respectively, the linear expression (Equation (6)) of the pseudo-second-order kinetics model will result, as follows

(6) $$\frac{t}{q_{t}}=\frac{1}{k_{2}\times q_{e}^{2}}+\frac{t}{q_{e}}$$

The graphical representation of the mathematical function will consist of a straight line from which the adsorption rate constant of the pseudo-second-order (*k*_2_) can be calculated by using the slope of the straight line. 

The experimental adsorption data in the case of paracetamol and acetylsalicylic acid were fitted to the linear form of the kinetic models. The linear regression analysis was employed in order to validate the kinetic models.

The experimental equilibrium data were fitted by the consecrated isotherm models: Langmuir (Equation (7)), Freundlich (Equation (8)), and Redlich–Peterson (Equation (11)). The adsorption isotherms provide important information regarding the adsorption mechanism, the surface properties of the adsorbent, the affinity between the adsorbent and the adsorbate as well as the quantitative evaluation of the adsorption process performance [58,59,60,61].

The Langmuir isotherm is described by the equation

(7) $$q_{e\;}=\frac{q_{m}\;\times K_{L}\times C_{e}}{1+K_{L}\times C_{e}}$$

where, *q*_e_—the amount of solute adsorbed at equilibrium [mg/g]; *q_m_*—the maximum monolayer adsorption capacity at the formation of the molecular layer [mg/g]; *C_e_*—the equilibrium concentration of the solute [mg/L]; and *K_L_*—the Langmuir adsorption constant [L/mg]. 

The Freundlich isotherm is expressed as follows

(8) $$q_{e}=\;K_{F}\times C_{e}^{1/n}$$

where, *K_F_*—the Freundlich constant [mg^1−1/n^ L^1/n^ g^−1^], indicating the adsorption capacity of the adsorbent; n—a constant, indicating the intensity of adsorption (dimensionless).

The Langmuir and Freundlich equations allow the assessment of the adsorption capacity of the carbon surface magnetic matrix. The *q_m_* and *K_L_* constants can be obtained by linearization of Equation (7):

(9) $$\frac{C_{e}}{q_{e}}=\frac{1}{q_{m}\times K_{L}}+\frac{1}{q_{m}}\times C_{e}$$

By graphically representing the function a straight line will emerge from which the values of *q_m_* and *K_L_* constants can be calculated by using the slope and the intercept of the straight line. The Freundlich equation can also be expressed as a linear function:

(10) $$\ln q_{e}=\ln K_{F}+\frac{1}{n}\times \ln C_{e}$$

The graphical representation of the function will produce a straight line from which the n parameters and K_F_ constant can be calculated by using the slope and the intercept of the straight line, respectively. 

The Redlich–Peterson isotherm is an empiric isotherm that contains three parameters; this isotherm combines the characteristics of both Langmuir and Freundlich isotherms. The Redlich–Peterson isotherm is described below.

(11) $$q_{e}=\frac{K_{RP}\times C_{e}}{1+\alpha_{RP}\times C_{e}^{\beta }}$$

where, *K_RP_*—the Redlich–Peterson constant [L/g]; *α_RP_*—constant [L/mg]^β^; and *β*—an exponent ranging between 0 and 1. 

Equation (11) reduces to Henry’s Law when *β* = 0 and becomes the Langmuir isotherm when *β* = 1 [47].

The correlation coefficient (R^2^) was obtained by nonlinear regression in order to choose the most suitable isotherm model for the experimental equilibrium data.

## 4. Conclusions

The potential of using a Fe_3_O_4_@C matrix obtained by combustion method as adsorption material for the removal of paracetamol and acetylsalicylic acid was investigated. Results show that the as synthesized magnetic matrix is suitable for both paracetamol and acetylsalicylic acid removal; this being the first such study reported in the literature. 

Taking into account that at a specific dose both drugs become toxic, their removal from the biological/aqueous medium is imperative. It is well known that magnetite (Fe_3_O_4_) shows superparamagnetic properties and is biocompatible in specific conditions. In addition, activated charcoal is the most commonly used adsorbent in almost all cases of drug intoxication. 

Altogether, our synthesized matrix, based on both magnetite and activated charcoal, can be successfully used in the adsorption of various drugs from aqueous solution, wastewater and even biological medium. 

Besides the novelty offered by this study (two drugs were adsorbed onto a magnetite–core active charcoal–shell matrix), the magnetic properties of Fe_3_O_4_@C matrix are of great significance. Such properties allow these type of nanoparticles to be magnetically controlled efficiently, and they can also be reused in multiple adsorption–desorption cycles. These aspects represent important findings that can be used as various starting points for future studies. 

## Figures and Tables

**Table 1:** Elemental composition of the Fe_3_O_4_@C matrix.

Element	Wt%	At%	K-Ratio	Z	A	F
C k	50.48	72.62	0.1729	1.0502	0.3261	1.0003
O k	15.66	16.91	0.0276	1.0342	0.1705	1.0010
Fe k	33.86	10.47	0.3140	0.8872	1.0453	1.0000
Total	100.00	100.00				

**Table 2:** Kinetics parameters and the correlation coefficients (R^2^) for the paracetamol and acetylsalicylic acid adsorptions onto Fe_3_O_4_@C matrix.

Pseudo-Second-Order						Pseudo-First-Order	
Adsorbate	Conc. [mg/L]	k_2_·10^3^ [g·mg^−1^·min^−1^]	R^2^	q_e_ [mg/g]		R^2^	k_1_·10^3^ [min^−1^]
				Experimental	Calculated		
Paracetamol	50	22.94	0.99996	24.36	24.47	0.94914	11.37
Acetylsalicylic acid	100	3.56	0.99977	43.43	44.62	0.88386	10.38

**Table 3:** Isothermal parameters and correlation coefficients for paracetamol and acetylsalicylic acid adsorption, respectively, on 2 g/L mass of Fe_3_O_4_@C matrix.

Analgesic Drug	Isotherm Model	Parameter	
**Paracetamol**	Langmuir	K_L_ [L mg^−1^]	0.01384
		q_m_ [mg g^−1^]	142.011
		R^2^	0.95703
		χ^2^	107.22
	Freundlich	K_F_ [mg^1−(1/n)^L^1/n^g^−1^]	13.48712
		n	2.77126
		R^2^	0.9838
		χ^2^	40.41
	**Redlich–Peterson**	K_RP_ [L g^−1^]	8.94606
		α_RP_ [(L mg^−1^)^β^]	0.41013
		β	0.71298
		**R^2^**	**0.98593**
		χ^2^	35.10
**Acetylsalicylic acid**	Langmuir	K_L_ [L mg^−1^]	0.00452
		q_m_ [mg g^−1^]	234.0139
		R^2^	0.95008
		χ^2^	161.44
	**Freundlich**	K_F_ [mg^1−(1/n)^L^1/n^g^−1^]	6.64178
		n	1.91846
		**R^2^**	**0.99421**
		χ^2^	18.73
	Redlich–Peterson	K_RP_ [L g^−1^]	479009.3512
		α_RP_ [(L mg^−1^)^β^]	72115.6797
		β	0.47876
		R^2^	0.99228
		χ^2^	24.97

**Table 4:** Comparison of the removal efficiency of paracetamol using different adsorbents.

Adsorbent	Obtained/Acquisition Mode	Adsorbent Quantity [g L^−1^]	Adsorption Condition	Equilibrium Time	Maximum Adsorption Quantity [mg g^−1^]	References
Fe_3_O_4_@C matrix	combustion method	2	T = 25 °C pH = 3	1 h	141.99	this study
Reduced activated carbon	obtained from heated granulated carbon at 900°C	4	T = 25 °C pH = 7	48 h	245.7	[16]
Activated carbon	obtained by various biological precursors materials	1	T = 30 °C pH = 3	20 min	99.42	[52]
Activated carbon	purchased (Ever Gainfull Enterprise Sdn. Bhd)	1	T = 40 °C pH = 2	2 h	92.17	[53]
Fe/N-CNT/β-CD nanocomposite	microwave-assisted method	1	T = 25 °C pH = 7	30 min	75.2	[50]
N-CNT/β-CD nanocomposite					41	

**Table 5:** Comparison of the removal efficiency of acetylsalicylic acid using different adsorbents.

Adsorbent	Obtained/Acquisition Mode	Adsorbent Quantity [g L^−1^]	Adsorption Condition	Equilibrium Time	Maximum Adsorption Quantity [mg g^−1^]	References
Fe_3_O_4_@C matrix	combustion method	2	T = 25 °C pH = 3	2 h	234.02	this study
Banana peel bioadsorbent	obtained from the banana silver fruit	6	T = 25 °C pH = 7	15 min	2.29	[49]
Fe/N-CNT/β-CD nanocomposite	microwave-assisted method	1	T = 25 °C pH = 2	30 min	101.0	[50]
N-CNT/β-CD nanocomposite					71.9	
Activated carbon	purchased (Sigma-Aldrich)	0.15	T = 26 °C pH = 1.5	48 h	236.0	[51]
Sephabeads SP 206 Polymer	purchased	0.84	T = 20 °C pH = --	150 min	45.2	[54]
Sephabeads SP 207 Polymer		0.72			81.6	
